# Effects of alcohol abstinence on glucose metabolism in Japanese men with elevated fasting glucose: A pilot study

**DOI:** 10.1038/srep40277

**Published:** 2017-01-09

**Authors:** Takashi Funayama, Yoshifumi Tamura, Kageumi Takeno, Minako Kawaguchi, Saori Kakehi, Takahiro Watanabe, Yasuhiko Furukawa, Hideyoshi Kaga, Risako Yamamoto, Akio Kanazawa, Yoshio Fujitani, Ryuzo Kawamori, Hirotaka Watada

**Affiliations:** 1 Department of Metabolism & Endocrinology, Juntendo University Graduate School of Medicine, 2-1-1 Hongo, Bunkyo-ku, Tokyo 113-8421, Japan; 2 Sportology Center, Juntendo University Graduate School of Medicine, 2-1-1 Hongo, Bunkyo-ku, Tokyo 113-8421, Japan; 3 Center for Therapeutic Innovations in Diabetes, Juntendo University Graduate School of Medicine, 2-1-1 Hongo, Bunkyo-ku, Tokyo 113-8421, Japan; 4 Center for Molecular Diabetology, Juntendo University Graduate School of Medicine, 2-1-1 Hongo, Bunkyo-ku, Tokyo 113-8421, Japan

## Abstract

It has been demonstrated that moderate alcohol consumption provides protection against the development of type 2 diabetes. However, several other reports suggested that moderate alcohol intake may increase the risk of type 2 diabetes in non-obese Japanese. The aim of present study was to investigate the effect of 1-week alcohol abstinence on hepatic insulin sensitivity and fasting plasma glucose (FPG) in non-obese Japanese men. We recruited 8 non-obese Japanese men with mildly elevated FPG and drinking habits alcohol (mean frequency; 5.6 ± 2.5 times/week, mean alcohol consumption; 32.1 ± 20.0 g/day). Before and after the 1-week alcohol abstinence, we used the 2-step hyperinsulinemic-euglycemic clamp to measure endogenous glucose production (EGP) and insulin sensitivity (IS) in muscle and liver. One-week alcohol abstinence significantly reduced both FPG by 7% (from 105.5 ± 11.7 to 98.2 ± 7.8 mg/dl, *P* < 0.01) and fasting EGP by 6% (from 84.1 ± 4.2 to 77.6 ± 1.6 mg/m^2^ per min, *P* < 0.01), respectively. Two–step clamp study showed that alcohol abstinence significantly improved hepatic-IS, but not muscle-IS. In conclusion, one week alcohol abstinence improved hepatic IS and FPG in non-obese Japanese men with mildly elevated FPG and drinking habits alcohol.

The number of the patients with type 2 diabetes mellitus is rapidly growing worldwide and if effective methods to prevent its onset are discovered, we could reduce the health problem. Previous epidemiological studies showed that alcohol drinking provides protection against the development of type 2 diabetes. Indeed, a meta-analysis of 20 cohort studies^1^ demonstrated that moderate alcohol consumption reduces the onset of type 2 diabetes. However, it is noteworthy that in the above-mentioned meta-analysis study^1^, the subjects of 17 of the 20 studies were conducted in U.S. and European countries and only 3 studies were conducted in Japan (2/3) and Korea (1/3). Certainly, one of the 2 included Japanese epidemiological studies have shown that moderate alcohol intake is associated with decreased risk of type 2 diabetes^2^, however this was only observed in men with a BMI ≥ 22.1 kg/m^2^. On the other hand, both studies performed in Japan showed that high^2^ or moderate to high^3^ alcohol consumption was associated with an increased risk of type 2 diabetes among lean Japanese men (BMI ≤ 22.0 kg/m^2^). Consistently, systematic review demonstrated that moderate alcohol intake increases the risk of type 2 diabetes, especially in Japanese lean subjects^4^. Considering facts that East Asian subjects with normal BMI level (<25 kg/m^2^) can easily develop type 2 diabetes^5^ and large differences in the polymorphic distribution of alcohol-metabolizing enzymes have been reported between East Asian and Caucasian^6^, the effects of alcohol intake on type 2 diabetes might vary according to ethnicity.

As well as epidemiological studies showed various relation between alcohol consumption and the onset of type 2 diabetes, previous studies revealed various effects of alcohol on insulin sensitivity in peripheral tissues (mainly muscle) by using hyperinsulinemic euglycemic clamp, a gold standard method to evaluate insulin sensitivity. For example, one cross-sectional study demonstrated that alcohol consumption was positively correlated to insulin sensitivity in peripheral tissue^7^; however, alcohol intake was not related to insulin sensitivity in other cross-sectional studies^8^,^9^. Two intervention studies also demonstrated that chronic alcohol intake did not change insulin sensitivity in peripheral tissue^10^,^11^. In terms of hepatic insulin sensitivity, acute and chronic ethanol exposure induces hepatic insulin resistance in rats^12^,^13^; however, the effects of alcohol on hepatic insulin sensitivity has not been clarified in human. To evaluate hepatic insulin sensitivity by hyperinsulinemic euglycemic clamp in non-diabetic subjects, glucose tracer and low dose insulin infusion rate (IIR) (e.g. 10 mU/m^2^/min) are required. As mentioned above, several studies performed hyperinsulinemic euglycemic clamp to investigate the association between alcohol and insulin sensitivity^7^,^8^,^9^,^10^,^11^, no study did not apply hyperinsulinemic euglycemic clamp with glucose tracer and low dose insulin infusion rate.

Recently, we applied two step hyperinsulinemic (IIR = 10 and 20 mU/m^2^/min) euglycemic clamp study with glucose tracer to precisely evaluate hepatic insulin sensitivity in non-obese non-diabetic Japanese^14^,^15^. By matching the record of alcohol intake of each subjects, for the first time, we found that alcohol consumption negatively correlated with hepatic insulin sensitivity, but not with muscle insulin sensitivity^14^. In addition, our preliminary multiple regression analysis revealed that only alcohol consumption was independently correlated with hepatic insulin sensitivity^15^. Given that our data also showed that impaired hepatic insulin sensitivity correlated positively with fasting plasma glucose (FPG) level^14^, alcohol intake may impair hepatic insulin sensitivity and increases FPG in non-obese non-diabetic Japanese. However, the causal relationship between these factors has not been investigated yet.

Based on the above background, the present study was designed to investigate the effect of 1-week abstinence from alcohol beverage on hepatic insulin sensitivity and FPG in non-obese Japanese men with mildly elevated FPG. The results suggested that alcohol beverage abstinence improves both hepatic insulin sensitivity and FPG in those subjects.

## Results

Table 1 shows the clinical characteristics of the 8 subjects before and at the end of the 1-week intervention. The mean age of the subjects was 39.9 ± 4.7 and the mean BMI was 22.8 ± 1.3 kg/m^2^. Mean glucose, insulin, liver enzyme and lipid levels were within the normal range at baseline. The study subjects drank alcohol 5.6 ± 2.5 times/week and the mean consumption was 32.1 ± 20.0 g/day (Table 1). Binge drinker was defined as those who drink ≥42 ml of ethanol (3 drinks) per occasion (regardless of frequency)^16^ and 6 in 8 subjects were binge drinkers in the present study. In terms of type of alcohol, 60% was from beer, 21% from wine, 13% from distilled spirits and 6% from Japanese sake or makgeolli. Ethanol is oxidized to acetaldehyde by alcohol dehydrogenase (ADH) and subsequently acetaldehyde is oxidized to acetate by aldehyde dehydrogenase (ALDH). It has been reported that only ~5% of Japanese have enzymatically inactive type ADH1B (*1/*1)^17^, compared with ~90% of Caucasians^18^. In addition, only ~55% of Japanese individuals have active ALDH2 (*1/*1)^17^, compared with ~99% of Caucasians^18^. We examined their genotypes of study subjects and found that 7 in 8 subjects had active type of ADH1B (*2/*2) and only 1 subject had ADH1B (*1/*2). In addition, 5 subjects had active type of ALDH2 (*1/*1) and 3 subjects had ALDH2 (*1/*2). This prevalence is similar to Japanese population. All subjects reported adherence to alcohol beverage abstinence for 1 week, while daily physical activity level and calorie intake, except for alcohol beverage, were maintained during this period (Table 1).

As shown in Table 1, 1-week alcohol abstinence was associated with slight reduction in visceral fat area and total body fat, probably due to loss of energy from alcohol beverages. FPG was significantly decreased by 6% after the intervention, while fasting serum insulin did not change (Table 1). In addition, HDL cholesterol and liver function tests were significantly lower and LDL cholesterol were tended to be lower after the intervention (Table 1).

**Figure 1:**
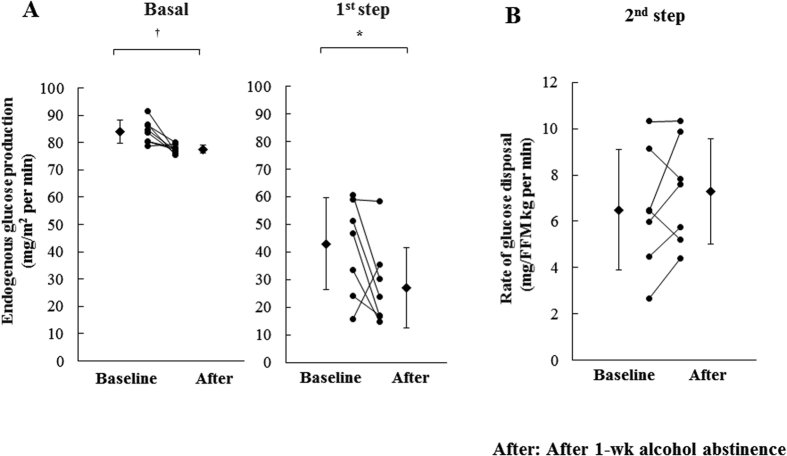
(**A**) Endogenous glucose production (EGP) at baseline and after 1 week alcohol abstinence. EGP levels were measured at fasting state and 1^st^ step during hyperinsulinemic-euglycemic clamp (n = 8). (**B**) Rate of disappearance (Rd) at baseline and after 1 week alcohol abstinence. Rd levels were measured at 2^nd^ step during hyperinsulinemic-euglycemic clamp (n = 7). Closed circle; individual data, Closed diamond; mean value. *p < 0.05, ^†^*p* < 0.01 vs. baseline. FFM; fat free mass.

The endogenous glucose production (EGP) at fasting state was significantly decreased by 7% after 1-week alcohol abstinence (from 84.1 ± 4.2 to 77.6 ± 1.6 mg/m^2^ per min, *P* < 0.01) (Fig. 1A). Two–step hyperinsulinemic euglycemic clamp study showed a significant decrease in EGP level at 1^st^ step (by 37%, from 42.9 ± 16.6 to 27.1 ± 14.6 mg/m^2^ per min, *P* < 0.05) (Fig. 1A), while Rd at 2^nd^ step showed no significant change after the intervention (from 6.5 ± 2.6 to 7.3 ± 1.9 mg/fat free mass (FFM) kg per min, *P* = 0.27). (Fig. 1B). These findings suggest that 1-week alcohol abstinence specifically improved hepatic insulin sensitivity, not muscle insulin sensitivity and FPG in our subjects.

## Discussions

Until now, the effects of alcohol on hepatic insulin sensitivity has not been precisely evaluated in human. Very recently, we applied two step hyperinsulinemic euglycemic clamp method with glucose tracer to accurately measure hepatic insulin sensitivity in non-obese non-diabetic Japanese and, for the first time, we found that alcohol consumption negatively correlated with hepatic insulin sensitivity, but not with muscle insulin sensitivity^14^,^15^. To investigate the causal relationships, the present study evaluated the effects of 1-week alcohol abstinence on glucose metabolisms in 8 non-obese Japanese men with mildly elevated FPG. The results demonstrated that 1-week alcohol abstinence improved insulin sensitivity in liver, but not in muscle, and reduced FPG level. The results are consistent with the cross-sectional study and suggest that alcohol abstinence has beneficial effect on glucose metabolism in non-obese Japanese men with mildly elevated FPG.

In the present study, we did not include a control group, thus we cannot exclude the possible influence of factors other than alcohol abstinence on the result. However, the subjects were carefully instructed to maintain other life styles, such as food intake and daily physical activity, and these parameters were almost stable during the intervention. Thus, we suppose that at least these parameters did not greatly contribute to changes in hepatic insulin sensitivity and FPG levels in the present study.

On the other hand, cutting the calorie intake from alcohol beverages even only for 1 weeks decreased adiposity modestly, but significantly and this decrease may contribute to improvement of hepatic insulin resistance. Indeed, it has been shown that visceral fat accumulation is closely associated with hepatic insulin resistance in type 2 diabetes^19^ and 8% body weight reduction improved FPG and hepatic insulin resistance in obese type 2 diabetic patients^20^. However, these studies generally included type 2 diabetic patients with obesity and the role of visceral fat accumulation on hepatic insulin resistance in non-obese non-diabetic subjects may be different from those subjects. Indeed, our previous study showed that visceral fat area was negatively correlate to muscle insulin sensitivity, but not to hepatic insulin sensitivity in non-obese non-diabetic Japanese^14^. In addition, we found only a modest decrease in visceral fat area in the present study. From these, this modest decrease in visceral fat might not greatly contribute to improvement of hepatic insulin resistance in the present study. Further study with control subjects who also decreased caloric intake to the same extent may clarify improved glucose metabolism was due to alcohol abstinence or calorie loss by the intervention.

Other possible mechanisms are that alcohol may directly impair hepatic glucose metabolism. It has been shown that insulin signaling was impaired by acute alcohol exposure in *in vitro* and *in vivo* study^21^. In addition, other studies demonstrated that alcohol induced hepatic insulin resistance, although this change was not accompanied by impaired insulin signal transduction in liver^12^,^13^. Interestingly, hepatic insulin resistance by alcohol was due to impaired hypothalamic insulin action to suppress hepatic glucose production^13^. Furthermore, other previous studies reported the possible role of oxidative stress in the pathogenesis of hepatic insulin resistance^22^,^23^. In terms of oxidative stress by alcohol, gamma-glutamyl transferase (γ-GTP) works as an antioxidant and γ-GTP overexpression may represent an adaptive response to persistent oxidative stress^24^. Thus, one can assume that γ-GTP could be associated with hepatic insulin resistance. In fact, our previous study found negative correlation between hepatic insulin sensitivity and γ-GTP and we also observed decreased γ-GTP levels by alcohol abstinence. Although variable mechanism could be involved in hepatic insulin resistance by alcohol intake, these experimental results obviously support that the improved hepatic insulin sensitivity could be due to the release from such effects by alcohol abstinence.

The present study, together with our recent cross-sectional study^14^,^15^, suggested the association among alcohol consumption, hepatic insulin resistance and FPG in non-obese, non-diabetic Japanese men. However, given that there are large differences in the distribution of polymorphic ethanol-metabolizing enzymes between Asian and Caucasian^17^,^18^, our results cannot be generalized to other population. Indeed, the prevalence of ADH1B and ALDH2 of the subjects in this study was similar to those reported in Japanese population. It has been reported that differences in these polymorphic ethanol-metabolizing enzymes influence FPG level^25^, glycemic control^26^, insulin level^27^ and risk for diabetes^28^. Thus, we speculate that differences in the distribution of polymorphic ethanol-metabolizing enzymes between Asians and Caucasians may be responsible for the differences in the relation between alcohol intake and hepatic insulin sensitivity. Further studies are required to test this hypothesis.

There are several limitations in the present study. First, sample size is small. Second, the study subjects were non-obese Japanese men as well as our recent cross sectional study^14^,^15^, thus the effect of alcohol abstinence may be different in other populations. Third, the study did not include a control group, and thus we cannot exclude the possibility that other factors may have influenced the results. However, we carefully monitored the life styles, at least food intake and physical activity, and these factors were almost fixed during the intervention. Fourth, we included only men with moderately elevated FPG levels and 75% of study subjects were binge drinkers. Moderate to high^3^ alcohol consumption was associated with an increased risk of type 2 diabetes among lean Japanese men (BMI ≤ 22.0 kg/m^2^), but not women. Both elevated FPG^29^ and binge drinking^16^ are reported as risk factor of type 2 diabetes. The effect of alcohol abstinence might be well observed in such limited cases.

In conclusion, 1-week alcohol abstinence improved hepatic insulin sensitivity and FPG in non-obese Japanese men with mildly elevated FPG. Further randomized control trials in large number of subjects are required to confirm the presented data.

## Research Design and Methods

### Study subjects

Eight non-obese, non-diabetic Japanese men (BMI < 25 kg/m^2^) were recruited in this study. The inclusion criteria were: (1) age 25–50 years, (2) mildly elevated FPG (≥95 but <126 mg/dl), (3) alcohol drinking of more than 3 days per week. The following exclusion criteria were applied: (1) history of serious disease, (2) current disease or under treatment, (3) history of allergy to local anesthetics, (4) alcoholism, (5) heavy alcohol drinking (>110 g/day), (6) contraindication for magnetic resonance imaging (MRI) analysis, (7) unfit for the study judged by medical doctor. All subjects gave written informed consent to the study, which was approved by the Ethics Committee of Juntendo University. This study was performed in accordance with the principles outlined in the Declaration of Helsinki and registered with the Japan Clinical Trials Registry (UMIN-CTR000009537).

### Study design

This study was an open-label, non-randomized, single-arm study to investigate the effect of 1-week abstinence from alcohol beverage on glucose metabolism. Regular exercise was prohibited from 10 days before the first day of the study and the mean daily physical activity levels from 10 to 3 days before the first day of the study were estimated using an ambulatory accelerometer (Lifecorder; Suzuken, Nagoya, Japan). We also assessed energy intake by using dietary recording during the same period. Then, all subjects were asked to keep their daily physical activity at mean daily physical activity level ±10% throughout the study, which was monitored by accelerometer.

For 3 days before the first day of the study, the subjects were instructed to consume weight maintained standard diet (25% fat, 55% carbohydrate, 20% protein), which was provided as packed meal only on the day before the clamp study. Alcohol intake was prohibited from the day before the first study day. Then, we performed baseline evaluation at overnight fasting state. Total body fat content, intramyocellular lipid (IMCL) in the right tibialis anterior (TA) muscles, intrahepatic lipid (IHL) of segment 6 of the liver, and intra-abdominal and subcutaneous fat were measured. Then, 2-step hyperinsulinemic euglycemic clamp study with glucose tracer was performed to evaluate insulin sensitivity in muscle and liver, respectively. After baseline evaluation, all subjects were prohibited to drink alcohol beverages for 1 week. To monitor alcohol abstinence, subjects were told to check alcohol level before sleeping by a self-breathalyzer (TANITA, Tokyo, Japan). The subjects were instructed to maintain dietary intake similar to before alcohol abstinence. At the end of the 1-week alcohol abstinence period, all subjects repeated the same evaluations/measurements conducted at baseline. The main primary outcome of this study was fasting plasma glucose and insulin sensitivity in liver.

### Biochemical tests

Serum lipids (total cholesterol, HDL cholesterol, LDL cholesterol, free fatty acid (FFA) and triglyceride) and liver function tests (aspartate aminotransferase (AST), alanine aminotransferase (ALT) and gamma-glutamyl transferase (γ-GTP)) were measured by enzymatic methods and UV methods, respectively (SRL Inc., Tokyo). Serum adiponectin concentrations were measured by enzyme-linked immunosorbent assay (Daiichi Pure Chemicals, Tokyo).

### Genotyping

Genomic DNA was extracted from peripheral blood cells using a DNA extraction kit (QIAamp DNA Blood Kit; Qiagen, Tokyo, Japan). The ADH1B (rs1229984) and ALDH2 (rs671) genotypes were assayed by polymerase chain reaction–restriction fragment length polymorphism analysis as described previously^18^.

### Proton magnetic resonance spectroscopy

IMCL and IHL were measured after overnight fast as described previously^30^,^31^. Briefly, IMCL of the right TA muscle and IHL of segment 6 in the liver were measured by ^1^H-MRS using a knee coil and a whole body coil, respectively (VISART EX V4.40, Toshiba, Tokyo). Voxels (1.2 × 1.2 × 1.2 cm^3^ for muscle and 2 × 2 × 2 cm^3^ for the liver) were positioned in the TA muscle or liver, avoiding visible interfascial fat and blood vessels, and the voxel sites were carefully matched at each examination. Imaging parameters were set as follows; repetition time 1500 ms, echo time 136 ms (muscle) or 10 ms (liver), acquisition numbers 192 for muscle and 8 for liver, and 1,024 data points over a 1,000-kHz spectral width. After examination, resonance was quantified by reference to the methylene signal intensity (S-fat), with peaks being observed at ~1.25 ppm in muscle and at ~1.3 ppm in the liver. IMCL in right TA muscle was quantified by S-fat and the creatine signal at 3.0 ppm (Cre) as the reference, and was calculated as a ratio relative to Cre (S-fat/Cre) (n = 8). IHL was quantified by S-fat and H_2_O at ~4.7 ppm as the internal reference, and calculated as a percentage of H_2_O + S-fat [S-fat × 100/(H_2_O + S-fat)] as described previously^30^,^31^ (n = 5).

### Intra-abdominal and subcutaneous fat

Intra-abdominal and subcutaneous fat areas were measured as described previously using MRI^31^. Briefly, T1-weighted trans-axial scans were obtained to determine intra-abdominal and subcutaneous fat in a region extending from 8 cm above to 8 cm below the fourth and fifth lumbar interspaces (16 slices, field of view 370 × 400 mm^2^, slice thickness 10 mm, breath-hold repetition time 6000 ms, echo time 78 ms). Intra-abdominal and subcutaneous fat areas at fourth and fifth lumbar interspaces were measured as described previously using specific software (AZE, Tokyo, Japan)^31^.

### Euglycemic hyperinsulinemic glucose clamp test

After an overnight fast, a two-step euglycemic hyper-insulinemic glucose clamp study was performed with an artificial endocrine pancreas (STG 22; Nikkiso, Shizuoka, Japan)^14^. Briefly, after securing an intravenous cannula in the forearm, [6,6-^2^H_2_]glucose was infused intravenously, followed by constant infusion of 2 mg/m^2^ body surface area (BSA) per min for 3-h (−180 to 0 min) to measure fasting EGP^32^. This was followed by primed insulin infusion (40 mU/m^2^ per min for 5 min followed by 20 mU/m^2^ per min for 5 min) and continuous insulin infusion at 10 mU/m^2^ per min for 3 hours (first step) (0 to 180 min)^33^,^34^. In the second step of clamp, we continuously infused insulin at 20 mU/m^2^ per min for 3 hours (180 to 360 min) after priming insulin infusion (80 mU/m^2^ per min followed by 40 mU/m^2^ per min; each lasted 5 min). We used warming blanket for arterialization of hand vein and plasma glucose level in arterialized blood was maintained at ~95 mg/dl by a variable 20% glucose infusion containing ~2.5% [6,6-^2^H_2_]glucose. The infusion of [6,6-^2^H_2_]glucose was decreased by 75% of the initial infusion rate during first step and 85% of basal during second step to steadily maintain the plasma glucose enrichment. Blood samples were withdrawn for biochemical analysis at 10 min intervals during the last 30 min before the clamp and steady state periods of the first and second steps of the clamp. Enrichment of [6,6-^2^H_2_]glucose in plasma was measured by high-performance liquid chromatography with LTQ-XL-Orbitrap mass spectrometer (Therm Scientific, CA) as described previously^35^. Steady state equation was used to calculate the rates of EGP and Rd at each step^36^. We used the EGP suppression during the first step as an index of hepatic insulin sensitivity and rate of disappearance (Rd) of glucose during the second step as an index of muscle insulin sensitivity. However, we could not successfully measure Rd in 1 subject. Thus, in this study, we used EGP suppression in 8 subjects and Rd in 7 subjects for further analysis.

### Statistical analysis

All data are expressed as mean ± SD. Data that showed skewed distribution were log-transformed before analysis. Student’s *t* test was used for comparison of paired observations. Statistical significance was set at p < 0.05.

## Additional Information

**How to cite this article**: Funayama, T. *et al*. Effects of alcohol abstinence on glucose metabolism in Japanese men with elevated fasting glucose: A pilot study. *Sci. Rep.* **7**, 40277; doi: 10.1038/srep40277 (2017).

**Publisher's note:** Springer Nature remains neutral with regard to jurisdictional claims in published maps and institutional affiliations.

## Figures and Tables

**Table 1:** Clinical parameters measured at baseline and end of study.

	Baseline	End of study	*P* value
Body weight (kg)	68.6 ± 7.1	68.0 ± 6.5	0.20
Total body fat content (%)	22.8 ± 3.0	21.5 ± 3.4	0.03
Non-alcoholic calorie intake (kcal/day)	1911 ± 554	2007 ± 564	0.32
Alcohol beverage’s calorie intake (kcal/day)	280 ± 165	0 ± 0	< 0.01
Alcohol consumption (g/day)	32.1 ± 20.0	0 ± 0	< 0.01
Daily physical activity (METs·h)	3.8 ± 0.9	3.6 ± 1.5	0.51
Fasting plasma glucose [70–109] (mg/dl)	105.5 ± 11.7	98.2 ± 7.8	0.02
Fasting serum insulin [1.84–12.2] (μU/ml)	5.7 ± 2.9	5.4 ± 3.0	0.43
Aspartate aminotransferase [10–40] (IU/L)	25.3 ± 8.0	20.9 ± 4.3	0.08
Alanine aminotransferase [5–40] (IU/L)	35.6 ± 21	24.8 ± 9.9	0.04
γ-Glutamyl transferase [≤70] (IU/L)	55.4 ± 25	40.4 ± 15	0.04
Free fatty acid [140–850] (mmol/L)	391.3 ± 79.7	462.8 ± 129	0.13
Triglyceride [50–149] (mg/dl)	131.8 ± 40.0	119.0 ± 37.7	0.41
LDL cholesterol [70–139] (mg/dL)	121.1 ± 17.4	116.5 ± 17.6	0.09
HDL cholesterol [40–86] (mg/dL)	59.0 ± 18.3	50.6 ± 14.0	<0.01
Total adiponectin (μg/ml)	3.6 ± 1.3	3.5 ± 1.6	0.81
HMW-adiponectin (μg/ml)	1.2 ± 0.8	1.2 ± 0.8	0.88
Tumor Necrosis Factor -α (pg/ml)	7.5 ± 2.0	8.5 ± 2.5	0.06
high-sensitivity C-reactive protein (ng/ml)	403 ± 382	353 ± 210	0.74
Visceral fat area (cm^2^)	144.8 ± 34.3	132.8 ± 35.1	0.02
Subcutaneous fat area (cm^2^)	129.2 ± 44.8	125.9 ± 44.4	0.31
Intrahepatic lipid (%)	5.3 ± 6.8	5.7 ± 7.2	0.76
Intramyocellular lipid in TA (S-fat/Cre)	2.6 ± 1.5	3.1 ± 1.4	0.15

Data are mean ± SD of 8 subjects, except the rate of glucose disposal (n = 7), intramyocellular lipid in tibialis anterior muscle (TA) (n = 7) and intrahepatic lipid (n = 4). METs; metabolic equivalents, HMW; high-molecular weight, S-fat; methylene signal intensity.
